# Jasmonic acid is involved in the signaling pathway for fungal endophyte-induced volatile oil accumulation of *Atractylodes lancea* plantlets

**DOI:** 10.1186/1471-2229-12-128

**Published:** 2012-08-02

**Authors:** Cheng-Gang Ren, Chuan-Chao Dai

**Affiliations:** 1 Jiangsu Engineering and Technology Research Center for Industrialization of Microbial Resources, Jiangsu Key Laboratory for Microbes and Functional Genomics, College of Life Science, Nanjing Normal University, Nanjing, 210046, P.R. China

**Keywords:** *Atractylodes lancea*, Endophytic fungi, Volatile oil, Jasmonic acid, Medicinal herb

## Abstract

**Background:**

Jasmonic acid (JA) is a well-characterized signaling molecule in plant defense responses. However, its relationships with other signal molecules in secondary metabolite production induced by endophytic fungus are largely unknown. *Atractylodes lancea* (Asteraceae) is a traditional Chinese medicinal plant that produces antimicrobial volatiles oils. We incubated plantlets of *A. lancea* with the fungus *Gilmaniella* sp. AL12. to research how JA interacted with other signal molecules in volatile oil production.

**Results:**

Fungal inoculation increased JA generation and volatile oil accumulation. To investigate whether JA is required for volatile oil production, plantlets were treated with JA inhibitors ibuprofen (IBU) and nordihydroguaiaretic acid. The inhibitors suppressed both JA and volatile oil production, but fungal inoculation could still induce volatile oils. Plantlets were further treated with the nitric oxide (NO)-specific scavenger 2-(4-carboxyphenyl)-4,4,5,5-tetramethylimidazoline-1-oxyl-3-oxide potassium salt (cPTIO), the H_2_O_2_ inhibitors diphenylene iodonium (DPI) and catalase (CAT), and the salicylic acid (SA) biosynthesis inhibitors paclobutrazol and 2-aminoindan-2-phosphonic acid. With fungal inoculation, IBU did not inhibit NO production, and JA generation was significantly suppressed by cPTIO, showing that JA may act as a downstream signal of the NO pathway. Exogenous H_2_O_2_ could reverse the inhibitory effects of cPTIO on JA generation, indicating that NO mediates JA induction by the fungus through H_2_O_2_-dependent pathways. With fungal inoculation, the H_2_O_2_ scavenger DPI/CAT could inhibit JA generation, but IBU could not inhibit H_2_O_2_ production, implying that H_2_O_2_ directly mediated JA generation. Finally, JA generation was enhanced when SA production was suppressed, and vice versa.

**Conclusions:**

Jasmonic acid acts as a downstream signaling molecule in NO- and H_2_O_2_-mediated volatile oil accumulation induced by endophytic fungus and has a complementary interaction with the SA signaling pathway.

## Background

*Atractylodes lancea*, a member of the Compositae family, is a traditional Chinese medicinal plant [1,2]. Volatile oils from *A. lancea* show antimicrobial activities as well. These oils comprise active secondary metabolites, including the characteristic components atractylone, β-eudesmol, hinesol, and atractylodin [3]. Secondary metabolites, such as terpenes, flavonoids, and alkaloids, are believed to be involved in plant responses to many biotic and abiotic stresses [4-6]. Another plant defense response is the activation of multiple signaling events [7,8]. For example, jasmonic acid (JA) biosynthesis by plants is induced by pathogen infection and elicitor treatment [9], and salicylic acid (SA) is involved in activating distinct sets of defense-related genes [10], such as those that encode pathogenesis-related (PR) proteins [11]. Also, many signaling molecules have been revealed to be involved in secondary metabolism [12-14].

Endophytes can coexist with their hosts and have great potential to affect the hosts’ metabolism [15]; their effects on plant accumulation of medicinal components have received much attention recently [16,17]. Unlike pathogens, endophytic fungi do not cause strong hypersensitive reactions in the host. But long-term colonization can induce various kinds of metabolites to accrue in hosts [17,18]. How endophytic fungus-host interactions affect the accumulation of plant secondary metabolites is an intriguing issue.

Jasmonic acid is a well-characterized plant signaling molecule that mediates plant defense responses [19] by responding to microbial infection and elicitor treatment [20]. Kunkel *et al..*[21] found that fungal elicitor caused rapid increases in JA production, secondary metabolite biosynthetic gene expression, and secondary metabolite accumulation in many plants. Exogenous JA application enhanced gene expression of secondary metabolite biosynthetic pathways, while the fungal elicitor-induced secondary metabolite accumulation could be abolished by JA synthesis inhibitors [13]. Most plant defense responses are regulated by many signal molecules, and “cross-talk” among multiple signaling pathways is important in plant cell signal transduction networks [21]. An increasing number of studies have shown that these signals do not function entirely independently; rather, they are influenced the magnitude or amplitude of various other signals [22].

Although interactions between SA- and JA-mediated signaling pathways have been reported to enhance the expression of plant defense-related genes, studies on interactions between JA and multiple signaling pathways (nitric oxide, hydrogen peroxide, and SA) in mediating plant secondary metabolite accumulation are rare. In this work, we report that JA acts as a downstream signal of nitric oxide (NO)- and hydrogen peroxide (H_2_O_2_)-mediated volatile oil accumulation in *A. lancea* plantlets induced by endophytic fungus *Gilmaniella* sp. AL12. Furthermore, we reveal an unusual complementary relationship between JA and SA in mediating the biosynthesis of medicinal plant secondary metabolites.

## Methods

### Plant materials and treatments

Meristem cultures of *Atractylodes lancea* (collected in Maoshan, Jiangsu Province, China) were established according to Wang et al. [22]. The explants were surface sterilized and grown in MS medium [23] supplemented with 0.3 mg/L naphthaleneacetic acid (NAA), 2.0 mg/L 6-benzyladenine, 30 g/L sucrose, and 10% agar in 150 mL Erlenmeyer flasks. Rooting medium (1/2 MS) contained 0.25 mg/L NAA, 30 g/L sucrose, and 10% agar. All media were adjusted to a pH of 6.0 before being autoclaved. Cultures were maintained in a growth chamber (25/18°C day/night, with a light intensity of 3400 lm/m^2^ and a photoperiod of 12 h) and subcultured every four weeks. Thirty-day-old rooting plantlets were used for all treatments.

Reagents used as specific scavengers or inhibitors, including ibuprofen (IBU), nordihydroguaiaretic acid (NDGA), 2-(4-carboxyphenyl)-4,4,5,5-tetramethylimidazoline −1-oxyl-3-oxide potassium salt (cPTIO), paclobutrazol (PAC), catalase (CAT), diphenylene iodonium (DPI), and 2-aminoindan-2-phosphonic acid (AIP), were purchased from Sigma-Aldrich (St. Louis, MO, USA). All exogenous signaling molecules and inhibitors were filtered using 0.22 μm diameter microporous membranes before use. Unless stated otherwise, inhibitors were applied 1 d before the application of signaling molecules or fungal inoculation.

### Fungal culture and treatments

The endophytic fungus AL12 (*Gilmaniella* sp.) was isolated from *A. lancea,* cultured on potato dextrose agar, and incubated at 28°C for five days [24]. Thirty-day-old plantlets were inoculated using 5-mm AL12 mycelial disks. An equal size of potato dextrose agar was used as a control. All treatments were conducted in a sterile environment and replicated at least three times to examine reproducibility.

### Measurement of H_2_O_2_ and NO

Thirty-day-old plants were incubated with fungal mycelia disks with or without inhibitors and were harvested 18 d later for determination of NO or H_2_O_2_. Inhibitors were 1.25 mmol L^-1^ cPTIO, 5.25 mKat L^-1^ CAT or 3 mmol L^-1^ DPI.

The generation of H_2_O_2_ by *A. lancea* plantlets was measured by chemiluminescence in a ferricyanide-catalyzed oxidation of luminol according to Schwacke and Hager [25], with modification. Leaf samples (1 g) were ground with 5 ml double distilled water. The homogenate was centrifuged at 13,000 g for 10 min, then 100 μL supernatant, 50 μL luminol (5-amino-2,3-dihydro-l,4-phthalazinedione), and 800 μL phosphate-buffered saline were mixed in a cuvette. The reaction was initiated with 100 μL K_3_[Fe(CN)_6_. To compare independent experiments, we used H_2_O_2_ as an internal standard. Fifty microliters of H_2_O_2_ (1 μM, freshly prepared) was added to the assay mixture containing 750 μL potassium phosphate buffer. One unit of H_2_O_2_ was defined as the chemiluminescence caused by the internal standard of 1 μM H_2_O_2_ per gram fresh weight.

The generation of NO was monitored using a NO detection kit (Nanjing Jiancheng Bio-engineering Inst., Nanjing, China) according to the manufacturer’s instructions. Leaf samples (1 g) were ground with 5 ml of 40 mM 4-(2-hydroxyethyl)-1-piperazineethanesulfonic acid (pH 7.2) and the homogenate was centrifuged at 14,000 g for 10 min. The supernatant was used for the NO assays. One unit of NO was defined as the absorbance variation caused by the internal standard of 1 μM NO per gram fresh weight.

At least 15 plantlets were assayed for each time point, and all treatments were performed in triplicate.

### Measurement of SA

Thirty-day-old plants were incubated with fungal mycelia disks with or without inhibitors and were harvested 18 d later for determination of SA. Inhibitors were 1 mmol L^-1^ PAC or 2.5 mmol L^-1^ AIP.

Salicylic acid was extracted followed the method of Verberne et al. [26], with some modifications. Five grams of whole plantlets was ground in liquid nitrogen and extracted in 2 ml methanol by sonication. After centrifugation at 14,000 g for 5 min, the supernatant was rotary evaporated, and the residue was resuspended in 250 μl of 5% trichloroacetic acid. The mixture was re-extracted with 800 μl acetic acid ester: cyclohexane (1:1 v/v). Finally, the organic phase was rotary evaporated until dry, dissolved with 600 μl HPLC mobile phase (methanol: 2% acetic acid: H_2_O, 50:40:10, v: v: v), and filtered with a 0.22-μm microporous membrane for determination.

The SA samples were quantified by HPLC using a reverse-phase column (Hedera Packing Material Lichrospher 5-C18, 4.6 × 250 mm, 5 μm, Bonna-Agela Technologies, Wilmington, DE, USA). The mobile phases flow rate was 1 ml min^−1^. Salicylic acid was detected at 217 nm at 25°C [14].

### Extraction and determination of volatile oils and JA

Thirty-day-old plantlets of *Atractylodes lancea* were incubated with 5-mm mycelial disks or PDA disks (control). Inhibitors (0.1 mmol L^-1^ IBU or NDGA) were added 1 d before fungal inoculation for JA determination.

Volatile oils were extracted from whole plantlets of *A. lancea*, including leaves and rhizomes (0.8–1.6% oil content in leaves, 2.2–3.4% in rhizomes), according to Zhang et al. [27]. The volatile oils were dried with anhydrous sodium sulfate and stored in dark glass bottles at 4°C for gas chromatograph (GC) analysis.

Following Juergen et al. [28], JA was extracted by grinding plant material (1 g) frozen in liquid nitrogen and extracting with H_2_O: acetone (30:70, v:v). Samples were store in dark glass bottles at −21°C for GC analysis.

GC determination was carried out using an 1890 series GC (Hewlett-Packard, Palo Alto, CA) equipped with a flame ionization detector. A DB-5 ms (30 m × 0.25 mm × 0.25 μm) column (Agilent, Santa Clara, CA, USA) was used with the following temperature program: column held at 60°C for 1 min after injection, increased by 10°C/min to 190°C, held for 2 min, increased by 5°C/min to 210°C, held for 2 min, increased by 10°C/min to 220°C, and held for 8 min. Nitrogen was used as carrier and the flow rate was 4 ml/min. Four main components of the volatile oils, atractylone, hinesol, β-eudesmol, and atractylodin, were quantitatively analyzed according to the method of Fang et al. [29]; their retention times were 14.57, 15.24, 16.21, and 22.18 min, respectively.

### Real-time quantitative RT-PCR analysis

Total RNA was extracted from leaves as described by Dong and Beer [30]. First-strand cDNA was synthesized from 1 μg of total RNA (PrimeScript RT Reagent Kit, Takara, Dalian, China). Real-time qPCR was performed using the DNA Engine Opticon 2 Real-time PCR Detection System (Bio-Rad, Hercules, CA, USA) and SYBR green probe (SYBR Premix Ex Taq system, Takara). The constitutively-expressed gene EF1α used as an internal positive control. The gene-specific primers used to amplify EF1α were 5′-CAGGCTGATTGTGCTGTTCTTA-3′ and 5′-TGTGGCATCCATCTTGT-3′ (241 bp product) and for *al*HMGR were 5′-GGTGAGAAAGGTCCTGAAA-3′ and 5′-CATGGTAACGGAGATATGAA-3′ (154 bp). The GenBank accession numbers of the *al*HMGR and EF1α genes are EF090602.1 and X97131, respectively.

The thermocycler program was as follows: 90 s at 95°C; 40 cycles of 30 s at 95°C, 30 s at 57°C, and 30 s at 72°C; and 5 min at 72°C. To standardize the data, the ratio of the absolute transcript level of the *al*HMGR genes to the absolute transcript level of EF1α was calculated for each sample of each treatment.

### Statistical analyses

Data were compiled using Microsoft Excel (Redmond, WA, USA). The values were represented as mean ± SD of three replicates for each treatment. Student’s *t*-test, one-way ANOVA, and Duncan’s multiple range test were used to identify significant differences (SPSS ver. 13.0, SPSS Inc., Chicago, IL, USA).

## Results

### Dependence of JA in fungus-induced volatile oil accumulation

**Figure 1:**
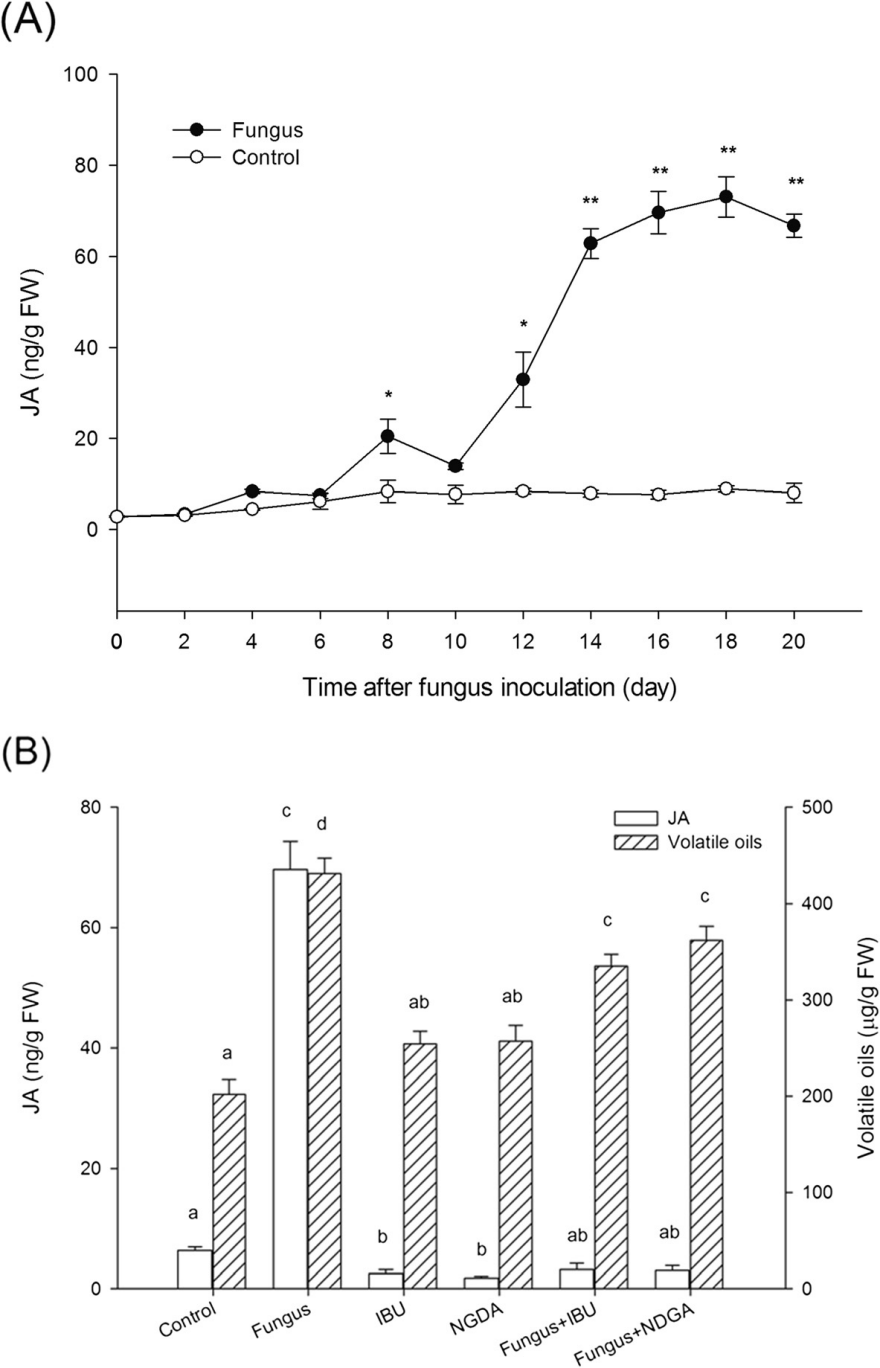
**Endophytic fungus-induced volatile-oil accumulation is dependent on JA generation.** Thirty-day-old plantlets of *Atractylodes lancea* were incubated with 5-mm mycelial disks or PDA disks (control). **(A)** Jasmonic acid production at 2-day intervals. Asterisks indicate significant differences from the control (0 d) (*t*-test; *, *P <*0.05; **, *P <*0.01). **(B)** Effects of JA inhibitors on endophytic fungus-induced volatile-oil accumulation after 18 d. Inhibitors (0.1 mmol L^-1^ IBU or NDGA) were added 1 d before fungal inoculation. Values are means of three independent experiments. Bars with different lower-case letters were significantly different (one-way ANOVA, Duncan’s multiple range test, *P <*0.05).

The JA contents of the plantlets increased significantly after endophytic fungus inoculation (Figure 1A), indicating that the fungus may trigger JA biosynthesis in the plantlets. Concurrently, the total amount of volatile oils increased significantly (Table 1). Both IBU and NDGA are inhibitors of the octadecanoid pathway that synthesizes JA and are usually applied in plant systems as JA-specific inhibitors [13]. To investigate whether JA was involved in the fungus-induced volatile oil accumulation, IBU and NDGA were applied; as shown in Figure 1B, both inhibitors suppressed not only the fungus-induced JA generation, but also the fungus-triggered volatile oil production. The results suggested that JA was important for fungus-induced volatile-oil synthesis in *A. lancea* plantlets. However, volatile oils in the *A. lancea* plantlets treated with both fungus and JA inhibitors could still accumulate, compared with the control, even though JA generation was lower than control (Figure 1B), implying that fungus-induced volatile oil synthesis is not solely dependent on the JA signaling pathway.

**Table 1:** **Accumulation of volatile oils by** ***Atractylodes lancea*** **over time**

**Components**	**Treatment**	**0 day**	**4 day**	**6 day**	**8 day**	**10 day**	**12 day**	**14 day**	**16 day**	**18 day**	**20 day**
Atractylone (μg/g)	Fungus	4.63 ± 1.41a	4.23 ± 0.74a	5.24 ± 0.94a	4.41 ± 0.67a	4.97 ± 0.56a	8.64 ± 1.19b	13.48 ± 1.54c	23.53 ± 2.76d	28.43 ± 1.54d	15.13 ± 0.93c
	Control	4.63 ± 1.27a	4.92 ± 1.02a	3.97 ± 0.42a	5.2 ± 0.55a	3.15 ± 0.75a	3.92 ± 0.48a	4.31 ± 0.39	4.71 ± 0.44a	5.17 ± 0.63a	5.6 ± 0.52a
Hinesol (μg/g)	Fungus	38.17 ± 4.32a	40.12 ± 3.82a	41.6 ± 4.93a	40.85 ± 5.63a	54.42 ± 4.23b	65.15 ± 5.28c	78.72 ± 6.63d	104.42 ± 8.23e	128 ± 9.42f	52.15 ± 4.45b
	Control	38.17 ± 5.36a	32.31 ± 3.52a	38.63 ± 3.78a	35.62 ± 3.29a	43.81 ± 4.22a	46.95 ± 3.04a	37.13 ± 6.27a	46.21 ± 3.23a	46.9 ± 3.32a	50.22 ± 5.24a
β-Eudesmol (μg/g)	Fungus	80.72 ± 11.37a	85.6 ± 6.01a	92.23 ± 6.43a	96.63 ± 6.48b	104.75 ± 6.12c	104.75 ± 6.06c	116.58 ± 6.19d	119.62 ± 6.25e	123.83 ± 8.07e	99.65 ± 4.18c
	Control	80.72 ± 10.75a	78.7 ± 8.32a	81.27 ± 8.53a	88.51 ± 7.95a	93.18 ± 8.28a	94.67 ± 8.05a	98.38 ± 5.04a	96.42 ± 8.15a	85.1 ± 8.18a	94.77 ± 7.84a
Atractylodin (μg/g)	Fungus	98.32 ± 14.53a	109.24 ± 11.31a	111.23 ± 12.95a	118.97 ± 12.74a	125.53 ± 17.85a	131.52 ± 12.34a	137.64 ± 15.31b	152.34 ± 12.92b	171.63 ± 12.04b	183.4 ± 12.39c
	Control	98.32 ± 12.75a	110.7 ± 10.61a	114.2 ± 7.76a	115.42 ± 8.23a	121.9 ± 10.28a	111.47 ± 12.71a	116.8 ± 10.07a	118.5 ± 10.63a	121.1 ± 10.75a	134.1 ± 10.68a
Total (μg/g)	Fungus	221.84 ± 31.63a	239.19 ± 21.88a	250.3 ± 25.25a	260.86 ± 25.52b	289.67 ± 28.76c	310.06 ± 24.87d	346.42 ± 29.67e	399.91 ± 30.15f	451.89 ± 31.07 g	350.33 ± 21.95e
	Control	221.84 ± 30.13a	226.63 ± 23.47a	238.07 ± 20.49a	244.75 ± 19.77a	262.04 ± 23.53a	257.01 ± 24.28a	256.62 ± 21.77a	265.84 ± 22.45a	258.27 ± 22.85a	284.69 ± 24.28a

Thirty-day-old plantlets were incubated with 5-mm mycelia disks or with an equal size of potato dextrose agar (control). Data are mean ± standard deviation (SD) of triplicate samples. Within each row, values followed by different lower-case letters were significantly different (one-way ANOVA, Duncan’s multiple range test, *P <*0.05).

### JA acts as a downstream signal of NO and H_2_O_2_ pathway

**Figure 2:**
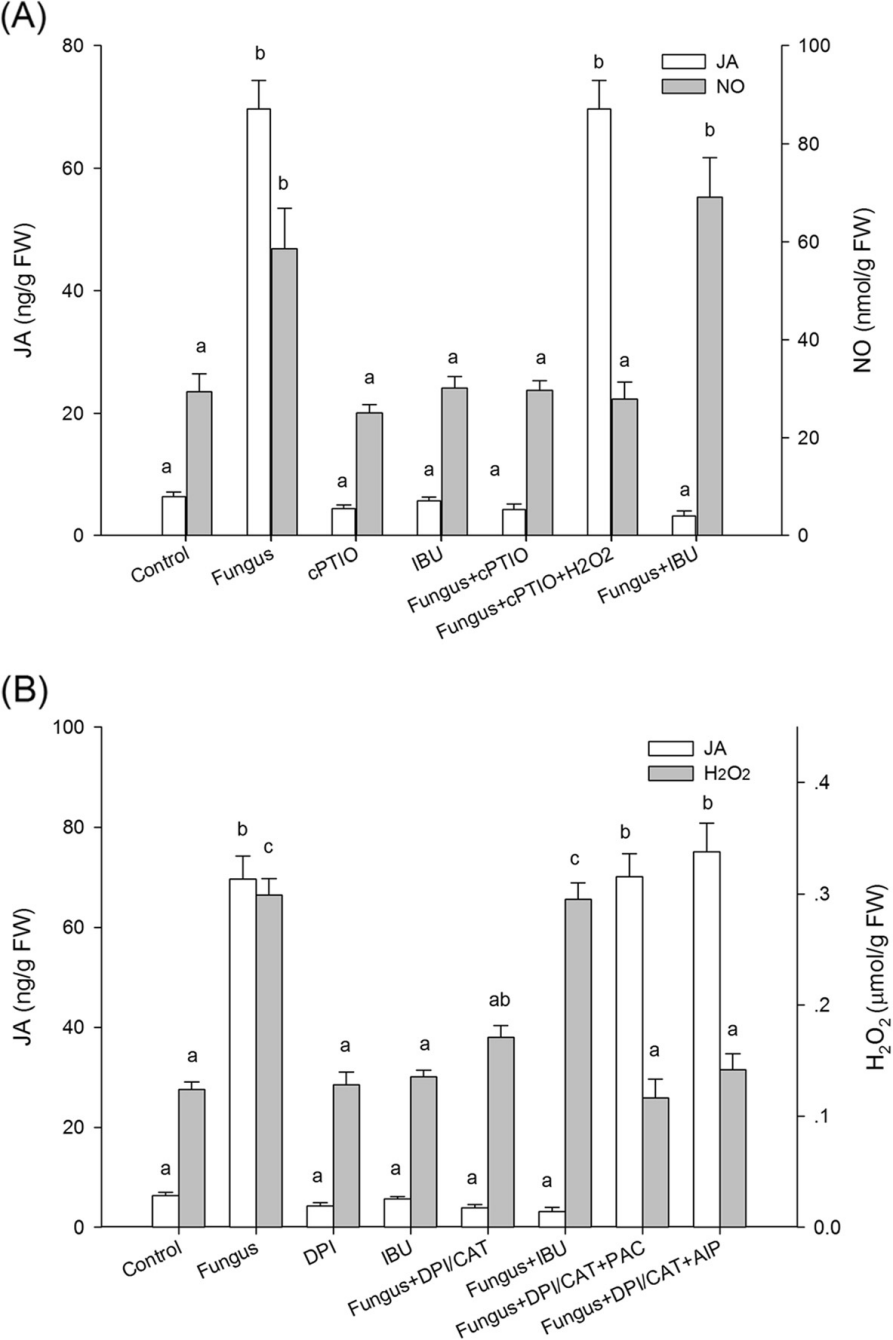
**Interactions between JA and NO or H**_**2**_**O**_**2**_**signaling pathways induced by endophytic fungus.** Thirty-day-old plantlets of *Atractylodes lancea* were incubated with 5-mm mycelia disks with or without inhibitors and were harvested 18 d later for determination of JA and NO or H_2_O_2_ concentrations. **(A)** Interactions between JA and NO pathways. Inhibitors were 1.25 mmol L^-1^ cPTIO, 0.1 mmol L^-1^ IBU, or 15 mmol L^-1^ H_2_O_2_. **(B)** Interactions between JA and H_2_O_2_. Inhibitors were 3 mmol L^-1^ DPI, 5.25 mKat L^-1^ CAT, 0.1 mmol L^-1^ IBU, 1 mmol L^-1^ PAC, or 2.5 mmol L^-1^ AIP. All inhibitors were added 1 d before fungus inoculation. Values are means of three independent experiments. Bars with different lower-case letters were significantly different (one-way ANOVA, Duncan’s multiple range test, *P <*0.05).

Previous results showed that JA is not the sole signaling pathway involved in fungus-induced volatile oil synthesis; NO, H_2_O_2_, and SA are also known to mediate this process in *A. lancea* plantlets [22]. To investigate a possible relationships between JA and one or more of these other pathways, *A. lancea* plantlets were treated with the NO-specific scavenger cPTIO, the membrane NADPH oxidase inhibitor DPI/CAT, the SA inhibitor PAC/AIP, IBU, and fungal inoculation. The NO scavenger cPTIO could inhibit JA production in inoculated plantlets, but IBU could not inhibit NO production (Figure 2A), showing that JA may act as a downstream signal of NO. Exogenous H_2_O_2_ could reverse JA suppression, implying that JA is mediated by NO though H_2_O_2_ in endophyte-induced volatile-oil accumulation. In addition, the H_2_O_2_ inhibitor DPI/CAT could inhibit JA production, but IBU could not inhibit H_2_O_2_ production with inoculation (Figure 2A). The one-way dependence of JA on H_2_O_2_ confirmed that H_2_O_2_ was the intermediary factor between JA and NO.

Paclobutrazol is an effective SA biosynthesis-related benzoic acid hydroxylase (BA2H) inhibitor [31] that also inhibits gibberellin biosynthesis [32]. Therefore, we also used AIP, a specific SA biosynthesis-related phenylalanine ammonialyase (PAL) inhibitor [33,34], to confirm that SA generation was suppressed. Interestingly, PAC and AIP could abolish the suppression of JA by DPI/CAT with fungus inoculation (Figure 2B). This result implied that the SA and JA signaling pathways were closely linked in endophyte-induced volatile-oil accumulation in *A. lancea* plantlets.

### Complementary interactions between JA and SA in fungus-induced volatile-oil accumulation

**Figure 3:**
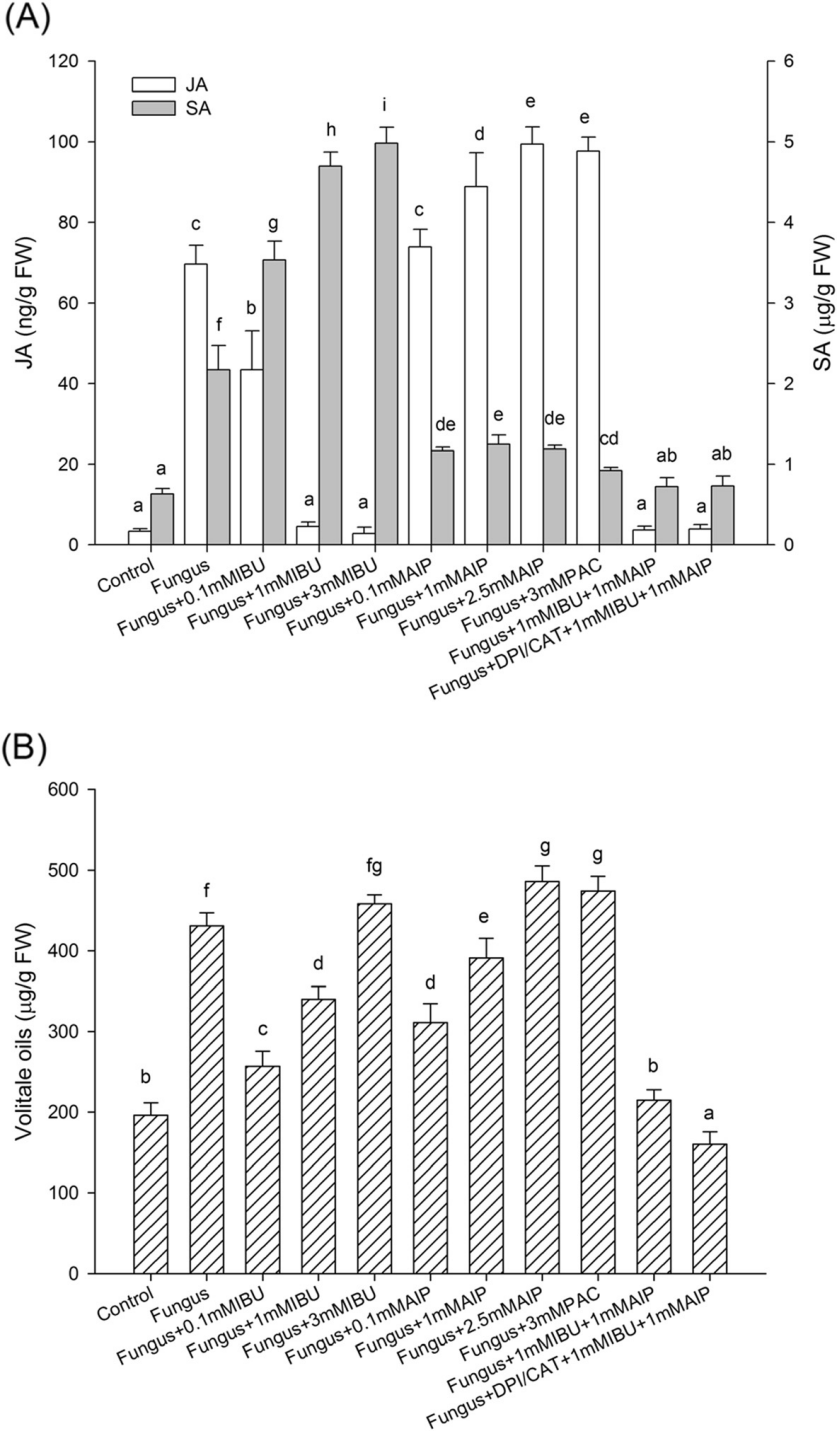
**Complementary interaction between JA and SA signaling pathways induced by endophytic fungus.** Thirty-day-old plantlets of *Atractylodes lancea* were incubated with 5-mm mycelia disks and 0.1, 1, or 3 mmol L^-1^ IBU; 3 mmol L^-1^ DPI; or 0.1, 1, or 2.5 mmol L^-1^ AIP and 3 mmol L^-1^ PAC. Plants were harvested 18 d later to determine JA and volatile oil levels. Inhibitors were added 1 d before fungus inoculation. **(A)** Interactions between JA and SA pathways. **(B)** Volatile oil production. Values are means of three independent experiments. Bars with different lower-case letters were significantly different (one-way ANOVA, Duncan’s multiple range test, *P <*0.05).

To further investigate the relationship between JA and SA, gradient concentrations of the JA-inhibiter IBU and the SA-inhibitors PAC and AIP were applied. As shown in Figure 3, the fungus-induced JA level of the plantlets decreased gradually as IBU concentration increased, but both SA accumulation and volatile oil content were enhanced as well, although the amounts did not exceed those obtained with fungal inoculation alone. Similarly, SA levels in plantlets were inhibited by 0.1, 1, and 2.5 mmol L^-1^ AIP and by 3 mmol L^-1^ PAC, whereas JA was enhanced significantly (Figure 3A). Volatile oil accumulation was enhanced by 2.5 mmol L^-1^ AIP and 3 mmol L^-1^ PAC (Figure 3B). The results suggested that JA may have a complementary interaction with SA to mediate fungal endophyte-induced volatile-oil accumulation. However, combining IBU and paclobutrazol could not completely inhibit volatile oil synthesis. We added the H_2_O_2_-inhibitor DPI/CAT to IBU and paclobutrazol, which reduced volatile-oil accumulation to the level of the control. The results suggested that H_2_O_2_, SA, and JA may work simultaneously in fungus-induced volatile-oil synthesis in *A. lancea* plantlets.

### Dependence of fungus-induced sesquiterpenoid production on JA production

**Figure 4:**
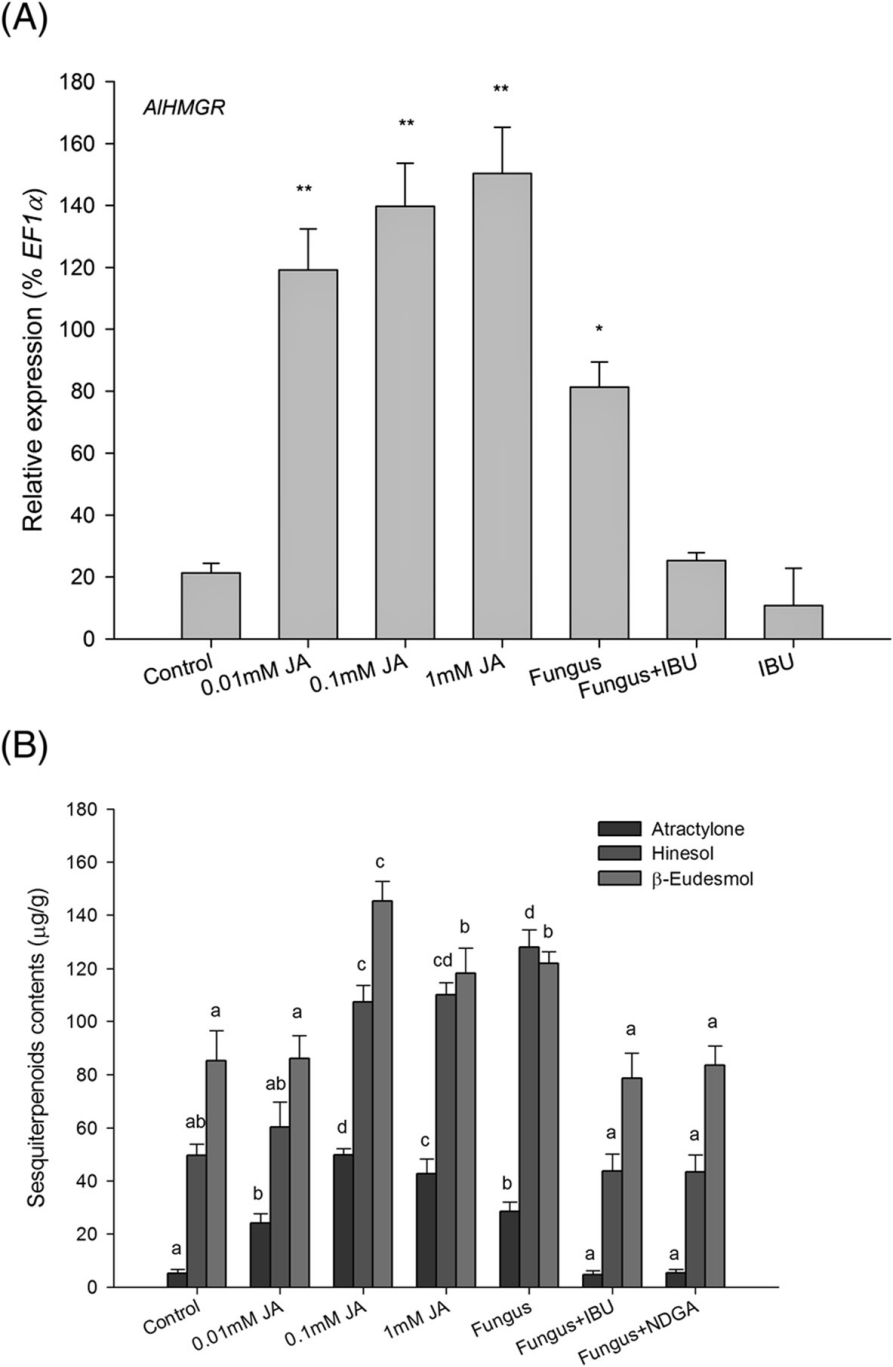
**Expression levels of HMGR genes and sesquiterpenoid accumulation responses to JA signaling pathway.(A)** Expression levels of EF1α and HMGR genes in response to JA determined by real-time qPCR and semi-qPCR analysis. Thirty-day-old plantlets of *Atractylodes lancea* were incubated with 5-mm mycelia disks; 0.01, 0.1, or 1 mmol L^-1^ JA; or 1 mmol L^-1^ IBU and harvested 18 d later for total RNA extraction and PCR analysis. Values are means ± SE (n = 3). Asterisks indicate significant differences (*t*-test; *, *P <*0.05; **, *P <*0.01). **(B)** Effects of JA on sesquiterpenoid accumulation. Plantlets were harvested after 18 d and evaluated for atractylone, β-eudesmol, and hinesol content. Values are means ± SE (n = 3). Bars with different lower-case letters were significantly different (one-way ANOVA, Duncan’s multiple range test, *P <*0.05).

The enzyme 3-hydroxy-3-methylglutaryl-CoA reductase (HMGR) catalyzes the conversion of HMG-CoA to mevalonate, which is the key step in the terpenoid biosynthesis pathway in plants [35,36]. We further investigated the possible mediating role of JA on HMGR gene expression. The results showed that exogenous JA could strongly stimulate HMGR gene expression (Figure 4A). Three sesquiterpenoid components of *A. lancea* volatile oils, atractylone, β-eudesmol, and hinesol, were all induced by JA and suppressed by IBU with fungal inoculation (Figure 4B).

**Figure 5:**
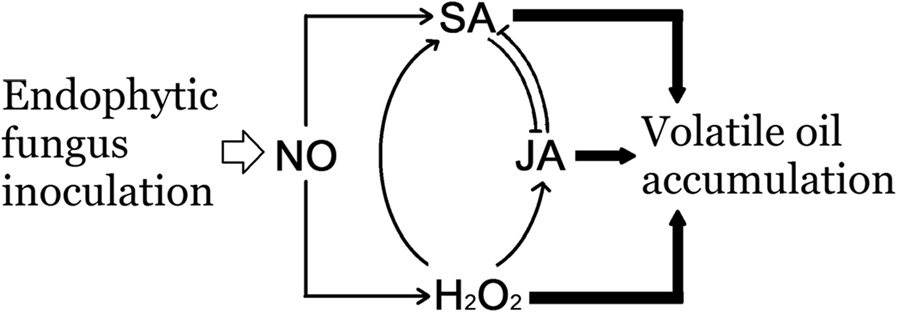
**Cross-talk between signaling pathways for volatile oil accumulation induced by endophytic fungal elicitor.** ‘’ indicates that the signal molecule was suppressed by specific inhibitors, while positive regulation is shown as ‘’.

## Discussion

Secondary metabolite accumulation is a common plant response to biotic or abiotic environmental stress, and secondary messengers are widely employed to mediate the accumulation of plant secondary metabolites. This work demonstrated that the fungus *Gilmaniella* sp. can induce JA production and promote the accumulation of volatile oils in host plantlets. As an important signal molecule, JA plays key roles in regulating the induction of volatile oils by the endophytic fungus. The specific inhibitors IBU and NDGA could block the JA signaling pathway and reduce the accumulation of related metabolites. Our previous study showed that NO, H_2_O_2_, and SA acted as signal molecules to mediate the accumulation of volatile oils in suspension cells of *A. lancea* caused by endophytic fungal elicitor [22]. Thus, the possible relationships between JA and other known signaling pathways in the accumulation of secondary metabolites were further investigated.

Cross-talk between different signal transduction pathways, as opposed to single signaling pathways, mediates gene expression and the production of secondary metabolites during plant defense responses [37,38]. Hydrogen peroxide has been reported to be a possible upstream signal for NO production in mung bean plantlets [39]. Nitric oxide also can mediated fungal elicitor-induced taxol biosynthesis in *Taxus chinensis* suspension cells through reactive oxygen signaling pathways, stimulate SA accumulation in tobacco cell cultures, and induce PAL expression via an SA independent pathway [31,40,41]. Moreover, our previous work demonstrated that NO mediates volatile oil accumulation induced by the fungus through SA- and H_2_O_2_-dependent pathways. Hydrogen peroxide can enhance SA production but does not act as upstream signal molecule [22]. The present work showed that endophytic fungus-induced JA was directly mediated by H_2_O_2_ and acted as a downstream signal molecule for both H_2_O_2_ and NO pathways.

In our study, JA had an unusual complementary interaction with the SA signaling pathway. Jasmonic acid is commonly postulated to act antagonistically on the SA signaling pathway and on the expression of SA-dependent genes [42,43]. Other studies have shown that SA is a potent suppressor of JA signaling pathways and JA-dependent defense gene expression in various pharmacological and genetic experiments [44,45]. In addition, both JA and SA are important signaling molecules in plant defense responses, such as the activation of distinct sets of defense-related genes and the development of systemic acquired resistance [21,46]. Our results showed that when JA biosynthesis was suppressed by the inhibitor IBU, accumulation of SA was enhanced to compensate for the loss of JA-mediating function in fungus-triggered volatile-oil production. Similarly, JA production/signaling could substitute for the SA pathway when SA accumulation was impaired.

## Conclusions

The value of medicinal herbs relies mainly on the accumulation of active pharmaceutical ingredients; low yield is the main challenge to producing high-quality herbs. In this work, we demonstrated that JA acts as a downstream signaling molecule in NO- and H_2_O_2_-mediated volatile oil accumulation induced by endophytic fungus and has a complementary interaction with the SA signaling pathway and clarified that HMGR gene expression was significantly stimulated by JA along with increasing sesquiterpenoid components. This information will help to better understand the relationships between fungal endophytes and their host plants. Furthermore, it also suggests strategies to improve the quality of medicinal herbs.

## Competing interests

The authors declare that they have no competing interests.

## Authors’ contributions

CGR designed experiment, analyzed data, and wrote the manuscript. CCD supervised the work and interpreted data with CGR. Both authors read and approved the final manuscript.
